# Folie en Famille: A Case Report of Shared Delusory Parasitosis

**DOI:** 10.1192/j.eurpsy.2022.2013

**Published:** 2022-09-01

**Authors:** A. Al Siaghy, Y. Zoghbi, M. Azeem

**Affiliations:** 1 Hamad Medical Corporation, Sidra Medicine, Psychiatry, Doha, Qatar; 2 Hamad Medical Corporation, Psychiatry, Doha, Qatar; 3 Sidra Medicine, Child And Adolescent Psychiatry, Doha, Qatar

**Keywords:** ekbom syndrome, Shared delusional disorder, Delusional parasitosis, folie en famille

## Abstract

**Introduction:**

Delusional parasitosis, first documented in 1946, is a rare psychiatric illness described as both a stand-alone diagnosis, as well as a secondary condition to an underlying psychiatric or medical pathology, or substance use. Interestingly, the fixed false belief of being infested has also been identified in partners of individuals with the disease, and in some cases the delusion permeated families and was thus given the name “folie en famille”.

**Objectives:**

To describe the first reported case of delusional disorder, somatic type, with similar delusional symptoms in the patient’s husband, in the State of Qatar.

**Methods:**

Patient and her husband were interviewed. Her file was reviewed for past history and medications.

**Results:**

34-year-old female with no past psychiatric history, 5 months post-partum, reported fixed beliefs of insect infestation in her baby’s skin, hers, and her husband’s, of 2 months duration. She reports a pruritic rash, and perceives proliferating insects in different life stages. The family relocated 5 times in 2 months. They bathe in vinegar several times a day to exterminate the insects. Husband mirrors her account of infestation with milder symptoms. Repeated medical investigations were insignificant. OCD, mood disorder, and other psychotic illnesses were ruled out.

**Conclusions:**

Delusional parasitosis presents a unique therapeutic challenge to psychiatrists. It is necessary to build rapport with patients, rule out comorbidities, and conduct randomized controlled trials to evaluate the effectiveness of psychotropic drugs in its treatment. In cases of shared delusions, identifying the primary patient is crucial for treatment of all the individuals that share the delusion.

**Disclosure:**

No significant relationships.

